# Integrating Cognitive Dysfunction Accommodation Strategies Into Behavioral Interventions for Persons on Medication for Opioid Use Disorder

**DOI:** 10.3389/fpubh.2022.825988

**Published:** 2022-02-10

**Authors:** Colleen B. Mistler, Christie I. Idiong, Michael M. Copenhaver

**Affiliations:** ^1^Department of Allied Health Sciences, University of Connecticut, Storrs, CT, United States; ^2^Institute for Collaboration on Health, Intervention, and Policy (InCHIP), University of Connecticut, Storrs, CT, United States

**Keywords:** opioid use disorder (OUD), behavioral interventions, cognitive dysfunction, accommodation strategies, qualitative analysis

## Abstract

**Background:**

Cognitive dysfunction is disproportionately prevalent among persons with opioid use disorder (OUD). Specific domains of cognitive dysfunction (attention, executive functioning, memory, and information processing) may significantly impede treatment outcomes among patients on medication for OUD (MOUD). This limits patient's ability to learn, retain, and apply information conveyed in behavioral intervention sessions. Evidence-based accommodation strategies have been integrated into behavioral interventions for other patient populations with similar cognitive profiles as persons with OUD; however, the feasibility and efficacy of these strategies have not yet been tested among patients on MOUD in a drug treatment setting.

**Methods:**

We conducted a series of focus groups with 25 key informants (10 drug treatment providers and 15 patients on MOUD) in a drug treatment program in New Haven, CT. Using an inductive approach, we examined how cognitive dysfunction impedes participant's ability to retain, recall, and utilize HIV prevention information in the context of drug treatment.

**Results:**

Two main themes capture the overall responses of the key informants: (1) cognitive dysfunction issues and (2) accommodation strategy suggestions. Subthemes of accommodation strategies involved suggestions about particular evidence-based strategies that should be integrated into behavioral interventions for persons on MOUD. Specific accommodation strategies included: use of a written agenda, mindfulness meditation, multi-modal presentation of information, hands-on demonstrations, and a formal closure/summary of sessions.

**Conclusions:**

Accommodation strategies to compensate for cognitive dysfunction were endorsed by both treatment providers and patients on MOUD. These accommodation strategies have the potential to enhance the efficacy of behavioral interventions to reduce HIV transmission among persons on MOUD as well as addiction severity, and overdose.

## Introduction

The opioid epidemic is an on-going public health crisis that continues to plague the US, as Opioid Use Disorder (OUD) diagnoses and overdose rates have exponentially increased in the past 10 years (1). The most common evidence-based medical treatment for OUD is medication for opioid use disorder (MOUD). MOUD is a comprehensive treatment strategy, often used in conjunction with behavioral interventions to reduce HIV and overdose risks. Common behavioral approaches include cognitive-behavioral therapy (CBT), motivational interviewing (MI), and contingency management CM; (2–4). Various psychoeducational counseling approaches are also commonly provided in the context of MOUD (5). Such approaches are often used to increase knowledge, motivation, and coping skills related to addiction and health risk reduction (6). By nature, these behavioral approaches often place cognitive demands on OUD patients, many of whom already experience cognitive dysfunction associated with drug use and related factors.

Cognitive dysfunction, commonly manifested as deficits in attention, executive functioning, and memory (7–11), can dramatically impede engagement and retention in drug treatment (12, 13). Researchers have identified cognitive dysfunction subcategories (including information and motivation constructs) that predict weakened HIV risk reduction behavioral skills and engagement in HIV prevention behaviors among persons on MOUD (14). The strategies used in behavioral interventions for OUD treatments may not be ideally tailored to meet the levels/forms of cognitive dysfunction among this population, thus limiting the efficacy of such interventions. Persons on MOUD often exhibit poorer decision-making and longer deliberation times due to cognitive dysfunction (15, 16). Cognitive dysfunction has been identified as a predictor of poor emotional perception among opioid-dependent individuals on MOUD (17). This poor emotional perception impacts patients ability to differentiate between positive and negative feelings of behavior, decreasing the ability to make rational decisions in regard to preventative behaviors (18). It can also adversely impact treatment outcomes (10, 14) such as treatment compliance, willingness to start and stay in treatment, attendance at behavioral intervention sessions, and lack of perspective on the benefits of treatment (19).

Patients with weaknesses in memory, attention, and communication may benefit from modified intervention content delivery and reinforcement (20). Cognitive dysfunction screening tools have been implemented among persons on MOUD, including the NIH Toolbox Cognition Battery (21) and Brief Inventory of Neurocognitive Impairment [BINI;(22)] to inform treatment protocols. However, cognitive dysfunction accommodation strategies tailored to persons on MOUD have not yet been assessed. Compensatory accommodation strategies (e.g., multimodal presentation of information, memory aids/reminders) have been shown to improve treatment outcomes (e.g., medication adherence, motivation) among other patient populations with cognitive dysfunction (23, 24). Most research on cognitive dysfunction accommodation strategies for behavioral interventions has been performed among people with cognitive profiles similar to those identified in persons on MOUD, including people with ADHD or post-TBI (5, 25, 26). There is a gap, however, in research on how such strategies may best be integrated with behavioral interventions in the context of drug treatment settings. Therefore, it is important to understand cognitive dysfunction among persons on MOUD and carefully match tailored accommodation strategies to limit the influence of such dysfunction in order to improve key treatment outcomes (27).

The aim of this study was to gain insight as to which accommodation strategies may be most useful in maximizing treatment outcomes of behavioral interventions for persons on MOUD. The following research question was used to guide data collection and analysis: “How can the experiences of drug treatment providers and people on MOUD inform HIV prevention efforts in a drug treatment setting?”

## Materials and Methods

### Study Design and Sample

The current study was designed as a phenomenological qualitative method design. Focus groups were utilized to help improve the development of HIV prevention programs in the context of a common drug treatment setting. Using a naturalistic approach, focus groups allowed researchers to collect information from a combined local perspective on this particular concept (28). We conducted separate sets of focus groups to gain insight from key informants- both providers and patients on MOUD. One focus group session was with drug treatment providers, and the other focus group session was with patients on MOUD.

The 10 treatment providers were all drug treatment counselors who provide direct care to patients on MOUD at APT Foundation, Inc. The credentials of these counselors include: Licensed Clinical Social Worker (LCSW), Licensed Professional Counselor (LPC), and Certified Drug and Alcohol Counselor (CDAC). All treatment providers facilitate group level behavioral intervention sessions to patients.

The 15 patients on MOUD all attended the APT Foundation, Inc. drug treatment center. The average age of patients was 51 years old and the average daily methadone dose was 83.6 mg. Four patients starting seeking treatment at the APT Foundation, Inc. as early as 2009, while a half of patients started being medicated at the drug treatment center after 2015. A majority of patients reported polysubstance use (93%) and one third of the participants reported having ever overdosed.

Focus groups were guided by an experienced facilitator who has worked with other collaborators to develop pre-determined open-ended questions, based on previous literature and historical outcomes about the content (29). All screening and focus groups were conducted in a private setting. The study protocol was approved by the Institutional Review Board at the University of Connecticut and received board approval from the APT Foundation, Inc.

### Data Collection

A convenience sample of participants was recruited via a variety of established methods in this setting to obtain a diverse sample of people on MOUD who are currently in drug treatment via in-clinic flyers, letters of invitation, and word of mouth recruitment. As the largest drug treatment provider in New Haven, with a census of over 4,500 patients, the APT Foundation, Inc. allowed for ample recruitment opportunities. Based on previous focus group research (29–31), including research on HIV prevention (32, 33), we enrolled 10 treatment providers and 15 patients in treatment from the research site. Recruitment occurred in June 2021 and all focus groups were conducted in July 2021.

Participants were reimbursed $25 for their attendance in each focus group. Participants were pre-screened for cognitive dysfunction using the Brief Inventory of Neurocognitive Impairment (22) BINI, and asked to identify their age, self-identified race and/or ethnicity, gender identity, engagement in drug- and sex-related HIV risk, HIV testing history, and past/present engagement in HIV prevention sessions. Individuals initially screened as eligible were invited to attend an in-person screening to confirm eligibility by meeting the following criteria: (a) at least 18 years or older, (b) in drug treatment and reported injection drug use in the past 30 days, (c) reported engagement in drug- and/or sex- related HIV risk behaviors, (d) are HIV negative, and (e) can communicate (read, write and speak) in English.

### Data Analysis

Using an inductive approach, in the context of Grounded Theory (34), the researchers analyzed the information using flexible coding (35, 36), to develop a theoretical basis for how cognitive dysfunction may impede participant's ability to retain, recall, and utilize HIV prevention information, in the context of a drug treatment setting. Audio recordings were transcribed and coded using NVivo software (37). Analytic memos and notes documented during focus groups were also used in developing an index of broad codes (38). Furthermore, analytic codes were constructed, based on the discussion points directly from participant's responses during the focus group sessions. During refinement, the researchers documented consistent trends across responses to further describe and apply in the context of other literature and real life applications of program development (39).

Using a bracketing approach, the researchers aimed to detach from preconceived concepts to avoid misinterpreting what the participants were saying about the content and to remain unbiased in the interpretation of their ideas. An audit trail of decisions, explaining the choices made throughout the study were also considered in the data analysis to maximize validity efforts taken by the researchers (40). Peer debriefing was utilized consistently throughout the research processes, and a second coder independently applied codes to the transcripts to enhance interrater reliability. Cohen's kappa coefficient was used to determine interrater reliability; substantial agreement was found between raters (*k* = 0.64). To maximize internal validity, we conducted a second round of focus groups with the same participants who engaged in the first round, as a member checking strategy, to ensure participants agreed with the information and themes we collected from them. This also provided the opportunity for participants to provide additional clarification about information that may have been brought up in their initial focus group session.

## Results

Utilizing focus group discussions, we coded two main themes to describe the responses of 25 key informants, including 10 treatment providers and 15 patients on MOUD. These two main themes were cognitive dysfunction and accommodation strategies. Subthemes of accommodation strategies were also identified, specifying which evidence-based cognitive dysfunction accommodation strategies were supported by key informants for integration into behavioral interventions in a drug treatment setting. Definitions and representative quotations are noted in Table 1.

**Table 1:** Emerging themes and subthemes of focus group discussions with drug treatment providers and patients on medication for opioid use disorder (MOUD).

**Theme/subtheme**	**Definition**	**Representative quotations**
Cognitive dysfunction^*^	Responses by key informants referring to memory loss, impaired literacy (reading and writing), or low levels of education related to years of opioid use. Includes responses by patients on MOUD recalling a low ability to process new information as they compared to their ability prior to drug use.	• → We definitely have people who can't read or write, so I'll help them and do it verbally with them. • → You get people that have memory deficiency or can't focus long enough to retain what you said. • → I mean maybe it comes down to, I mean maybe there are cognitive limitations, I'm sure there are. • → *I have difficulty remembering information*. • → I know some groups with low levels of education, so I condense my group and be mindful of how I can condense it and use some of the proverbs to make it make sense. I can't use this complicated verbiage.
Accommodation strategies^*^	Responses by key informants providing strategies to compensate for cognitive dysfunction to increase the ability to understand and retain information presented, optimal for a drug treatment setting	^**^See representative quotations listed in subthemes (i.e., written agenda, catchphrases, multimodal presentation, memory aids, mindfulness mediation, closure)
Written agenda^**^	Responses mentioning the benefit of using a written agenda to remain on task, guide discussion, and stay on time during sessions.	• → *I would like to see it because a lot of people like us get side tracked and at least we can get the leader of the group to say, ya know, we gotta move on to this. And we know what we covered*. • → Just to keep people engaged and some instances they get bored so keeping them on track is important. It makes them a part of the session the entire time, cause when I am just talking, they lose interest.
Catchphrases^**^	Responses referring to abbreviated terminology or simplified verbiage to keep patients engaged and improve retention of information.	• → Put it in different contexts and I paraphrase a lot and I use parallels. I tried to give an example like other similar thing, like a catch phrase. That's why I try to give like a popular saying that would equate to what the situation is, using a different scenario to reinforce the information.
Multimodal presentation^**^	Responses incorporating multiple methods of presenting information including: simplifying language, hands on demonstrations, visual aids and handouts.	• → I will bring in handouts, and it has to be kind of like a directed handout “1,2,3,4,5, something they can follow along with” Easy Verbiage because they just get bored, pictures, colors. • → I think anything you see or actually do, as opposed to just hearing, will help people remember a lot better. • → Sometimes the translations need to be simplified, and use more cultural terms on handouts would really make a difference. • → I think you would have to cut the steps down to like 3 steps, and be the safest way you can do it in the minimum amount of steps and showing them would be the most helpful. • → When the fentanyl came, I would give them a scenario and discuss the history of fentanyl with them with handouts. I would show them the differences in potency with the handout with visuals. • → *I think it's more useful to do it hands on, cause there are something I thought I was doing right, and I wasn't. I'm more of a hands on learner*. • → Some of my handouts are too extensive for them, so I break them down into portions and simplify them. its complex material so I like to have a handout with visuals. • → *For me, I like the videos, I like documentaries to learn, so that's just me*. • → It depends on how long the video or what it's about, cause something too long it's like “when is it gonna be over?” I would say nothing more than 15 min, anything longer than that is just overload and too much, especially if it's something they're not interested in. • → *A handout to debunk the myths*.
Memory aids^**^	Responses mentioning items that serve as reminders for daily tasks, taking medications, attending appointments, and/or behavioral sessions. Included are responses that refer to text message reminder to help patients remember appointments and medications.	• → *Honestly, anytime I can have something to remind me, it's helpful. I prefer weekly reminders though* • → *For me, it would be helpful and since I started the lady asked me if I wanted text reminders, and those are incredibly helpful*. • → *I think it's just so easy nowadays with cell phones. I Don't think people have much of an excuse to miss their dosing or their sessions, cause ya know especially if you're sending out 2 reminders in a day*. • → *I use the calendar function in my phone, like I even use that MyChart for my doctors' appointments*. • → We put it in the SMART program and set holds on their accounts to remind them. To help them get to their appointments, if we are aware of them. I don't know if we could do much more. So, if we have someone who we want to remind them to follow up with a mental health counselor, we can put it into the system and it will light up on the day. • → Text message reminder for appointments that are automated, like a day before and maybe an hour before or something like that. Yeah, some sort of clever system like that. • → *I say “this week, I have this and this and this” I make lists of what I have for the week. Most of it is in my head, but sometimes I write it down*.
Mindfulness meditation^**^	Responses encouraging and supporting meditation and breathwork as useful tools to decrease stimulation and increase learning.	• → Meditation is actually good, for the ones who like it, it really works. It relaxes you and puts you in a place you don't want to get up. • → So, I think it's all the distractions like if we could somehow clear a space and make this free from other stuff out and that sort of the environment I like to have. This is where you can clear your head. • → I'll just say let's do some cycles of breathing. And the whole group does it and it just makes them grounded and present, and they like it. • → *Ya know, when you have 10 things on your plate, I think it's good to take a breath to remind yourself why you're here*.
Closure^**^	Responses mentioning the benefit of summarizing the discussion that occurred in the session of the day to reinforce strategies and lessons taught.	• → *Having everyone say what they learned cause in the 1 h / 45 min group it's good to wrap up*. • → *They just like drilled it in and took their time and made sure we knew what we were doing before we left the group*. • → *I think it would be a good idea just to, at the end, reiterate what the point of being here was and it will help you know who was paying attention*. • → *I think it's good to go over, just so you can reinforce again. And different people have different things to talk about too so ya know I think it's good to end with everyone talking*.

*Standard font denotes responses from drug treatment providers and counselors. Italicized font denotes responses from patients*.

* *, Theme*;

** *, Subtheme*.

### Cognitive Dysfunction

Both drug treatment providers and patients on MOUD acknowledged the levels of cognitive dysfunction among the target population of patients on MOUD, and how it directly impacts their ability to stay engaged and recall information presented during group sessions. One provider stated how “memory is terrible, all around, because of the drug use; it does affect their memory, and the significance of the drugs they're using makes a difference on their memory.” Patients on MOUD also mentioned their inability to learn new information.

“I started using when I was 26 years old, and I still have the brain of my 26-year-old self. I haven't been able to learn and process new information since.”

When discussing patient's ability to remember information and prioritize their recovery, it was noted that “there's also a lot of contingencies; it depends on if their minds are fully working, and different factors.” Cognitive dysfunction was also mentioned in reference to a person's point in their recovery and its influence on motivation.

“It's all on a patient-to-patient basis. You cannot pinpoint what will work for everyone but it depends on where they are at in their recovery, so someone that's abstinent will respond differently to a different incentive than someone who is still actively using. But if you can identify the new clients, new in their recovery, everything you give they will take. They're eager like a kid.”

The use of worksheets during behavioral sessions was not supported due to cognitive limitations and low literacy levels, as exemplified by one provider: “We have clients that are functionally illiterate, so anything that needs to be filled out, I try to steer clear of those in general. Same with read alouds: they are always optional for me.” Similarly, patients also mentioned difficulties with reading and writing.

“I don't like writing because I don't like to have to think. I do have a hard time thinking, even though I've been clean for 6 years, still my mind still struggles to think about certain things, and it's sad, it really is.”

Providers also recommended the use of a brief screening strategy to determine patient's level of cognitive dysfunction, to adapt behavioral intervention strategies to meet the needs of those patients on demand. One provider said “I try to assess who I'm with in terms of comprehension.” Another provider thought of using clinical information to help screen participants level of cognitive dysfunction.

“I thinking about using like a questionnaire to figure out where people are at. I mean, if you see the client, you already have access to SMART [electronic health records], so if you have those tools, just a quick questionnaire, that you can fill out with the client and the answers will determine where the person is with mental health and with their addiction.”

### Accommodation Strategies

A variety of evidence-based cognitive dysfunction accommodation strategies were discussed with treatment providers and patients on MOUD. To increase patient's ability to pay attention, retain and recall information, participants highlighted that “any mixed method of presentation or use of handouts is helpful.” A multimodal presentation of information was supported, including the use of brief verbal presentation of information (<5 min of the facilitator talking), followed by the use handouts, videos, group discussion, and/or hands-on demonstrations. For example, one patient on MOUD indicated: “It is a combination of paper material and a video; I think it's always better to have both. Hands-on is always helpful.” A provider described it below:

“I would tell them about opioid receptors and I would use magnets to demonstrate how opioids transit in the brain. I had a little box and I had balls and I would use different color balls and say ‘this is how methadone affects the brain,' this is how the naloxone mimics the opioids, and use a square box with a round ball to show how the receptors react differently. A lot of people are more visual so that way, ya know, they're not verbal, they are visual.”

When discussing the use of video clips, it was consistently recommended to keep them short (<10–15 min) and to include a debrief of the topics discussed immediately following the video. For example, “Incorporating short clips in educational group, the more formats to present the information, the better.” **One** patient on MOUD noted how videos help to recall information by saying “you're watching something and, in your mind, you keep looking at it and thinking about it, and you think about it afterwards too.”

The use of simplified verbiage, catchphrases, and visuals were also recommended to be considered when developing handouts for patients to maximize their learning. Providers highlighted the importance of simplifying complex language for patients to better understand, as exemplified by: “so that they can relate to this broken-down version. I just really break it down a few pictures and there's like a lot of little things like catch phrases or slogans that catch their attention.”

Other accommodation strategies to help patients with cognitive dysfunction focus on the information being presented to them that were supported for use in a drug treatment setting included the use of a written agenda, memory aids, a short (1–2 min) mindfulness meditation and/or breathing activity at the beginning of sessions, and the use of a formal closure at the end of sessions. One patient on MOUD mentioned that “anything you see or actually do, as opposed to just hearing, will help people remember a lot better.”

Patients on MOUD supported the use of a written agenda to help keep them focused. For example, one patient indicated: “I think it's helpful, it helps me anyways and I can keep looking up there, and in my mind, I keep trying to remember what was put on that board and what we've talked about.” A provider also noted how a written agenda can reinforce goals and that patients “feel more accomplished” in striving to meet those goals when they can see what they covered in the sessions. A brief mindfulness meditation and/or breathing exercise was also supported to help improve patient's ability to focus on presented material. *One* provider mentioned doing “meditation here [the methadone clinic] for a year, 5 min for every group and it was always successful; they were more receptive to me and the information.” This was exemplified by:

“I think with my experience of running groups with these clients, I've been more surprised with how receptive the clients are to some of the things I was anxious about trying. The game group, meditation, I mean I've had big burly construction guys who love the meditation. So, I guess the only thing I would ask is to try! And if it doesn't work, adapt it for the next time.”

Patients on MOUD consistently noted the use of memory aids to help them remember to take their medications and attend appointments. One patient said “the text message reminder would really help me remember to take it [medication].” Both patients and providers supported the use of an automated reminder system to inform patients of upcoming treatment protocols, such as monthly check-ins with a counselor. One provider specifically mentioned that “it would be feasible” to integrate text message check-ins into treatment protocols. The use of a formal closure at the end of sessions to help patients recollect on the information and set realistic goals to focus on for the next session was also recommended. Participants noted that this gives them the opportunity to engage if they did not feel heard throughout the session; “I like this because sometimes you have a chance to talk, if you're quiet most of the time or don't like talking in front of other people, giving an opportunity to talk at the end is better.” A provider mentioned how they would “have them [patients] put out particular goals that they're trying to obtain, and confirm that that's what the goal is.” For example, one provider indicated:

“At the end of the session, we would do a review of the material to show you paid attention and learned something or have been affected by something that someone said. And you'll find commonality and people will build off of it and we discuss themes of the day and the topic of the group and the themes of the day. Being supportive and giving back to one another and just the whole concept of not being alone and sense of community or family.”

## Discussion

Research on the influence of cognitive dysfunction among people on MOUD is quite limited. Studies have shown that cognitive dysfunction may impede treatment outcomes among people on MOUD (10, 14); however, no studies have investigated which cognitive dysfunction accommodation strategies may be most useful and feasible for integration into behavioral interventions in a drug treatment setting. This study is novel in exploring the endorsement of specific accommodation strategies that may be optimal for maximizing treatment outcomes (medication adherence, retention in treatment, healthcare utilization) for persons on MOUD. Themes identified in this qualitative analysis indicated high rates of cognitive dysfunction among people on MOUD and support for the integration of certain specific accommodation strategies into behavioral HIV prevention interventions during routine drug treatment.

Key informants (drug treatment providers and patients on MOUD) endorsed specific accommodation strategies including: a brief mindfulness meditation at the beginning of sessions, memory aids to help patients remember information, going over group etiquette at the beginning of sessions, using agendas to keep participants on track during sessions, use of simple language and visuals in handouts, brief videos, hands on demonstrations, use of props and games, and use of closures focused on information reiteration and goal setting. Based on the success of these strategies in accommodating cognitive dysfunction among other patient populations with similar cognitive profiles, these strategies may enhance the efficacy of behavioral interventions by increasing patient's ability to learn, retain, and apply health behavior change information. Ideally, these strategies would be integrated into behavioral intervention sessions, and facilitated by drug treatment providers, to maximize participants' ability to engage in harm reduction behaviors.

Outcomes from this study can aid in informing future research to determine which of the proposed accommodation strategies may be most useful in compensating for (i.e., working around) the cognitive dysfunction often experienced by people on MOUD. Given the novelty of this area of inquiry, we recommend a series of future studies to investigate the impact of integrating these strategies on key outcomes among people on MOUD, including HIV prevention, overdose prevention, and retention in drug treatment. We recommend pilot work to test the feasibility of integrating these accommodation strategies into behavioral intervention sessions and to first determine the extent to which such strategies may boost outcomes. Although it is unclear whether certain individual or combinations of accommodation strategies might be most helpful among people on MOUD–given the diversity of cognitive profiles in this patient population-future research should examine key outcomes stemming from inclusion of a variety of combinations of strategies (e.g., Multiphase Optimization Strategy; MOST) (41). This research design would allow researchers to identify which combination of strategies most enhance patient's ability to process and utilize intervention content.

The present study provided analysis of what accommodation strategies were most supported by key stakeholders in a common type of drug treatment setting. We determined the preliminary acceptability of these accommodation strategies which provides an empirical foundation for further investigation/testing of selected strategies. While the outcomes from this study supported our concept of adapting accommodation strategies from other patient populations for use in a drug treatment setting, the efficacy of these strategies has not yet been examined among persons with MOUD.

## Conclusion

The economic and societal costs of OUD have continued to increase in the U.S. in the past 20 years (42). Nearly one million people have died from an overdose since 1999 (43), and over 100,000 people died from an overdose in the past 12 months (44). Additionally, people who inject drugs accounted for 10% of new HIV infections in 2018 (45). Treatment for OUD utilizes behavioral interventions to limit these negative outcomes among people on MOUD and to reduce a range of health risk behaviors. As researchers continue to investigate methods to improve patient's ability to engage in positive health behavior change, cognitive dysfunction is an often overlooked limitation to behavioral interventions in persons on MOUD. In focus group interviews, both treatment providers and patients on MOUD endorsed various accommodation strategies to compensate for cognitive dysfunction. These accommodation strategies have the potential to increase the efficacy of behavioral interventions to reduce overdose, death, and HIV transmission among persons on MOUD, and are worthy of further investigation in future work.

## Data Availability Statement

The raw data supporting the conclusions of this article will be made available by the authors, without undue reservation.

## Ethics Statement

The studies involving human participants were reviewed and approved by University of Connecticut Institutional Review Board. The patients/participants provided their written informed consent to participate in this study.

## Author Contributions

Material preparation, data collection, data analysis, and manuscript write-up were performed by CM and MC. CI contributed to the data analysis and final edits on the manuscript. The first draft of the manuscript was written by CM and all authors commented on previous versions of the manuscript. All authors contributed to the study conception and/or design. All authors have read and approved the final manuscript.

## Funding

This work was supported by grants from the National Institute on Drug Abuse (K24 DA051344 for MC) and the National Institute of Mental Health (T32 MH074387 for CM).

## Conflict of Interest

The authors declare that the research was conducted in the absence of any commercial or financial relationships that could be construed as a potential conflict of interest.

## Publisher's Note

All claims expressed in this article are solely those of the authors and do not necessarily represent those of their affiliated organizations, or those of the publisher, the editors and the reviewers. Any product that may be evaluated in this article, or claim that may be made by its manufacturer, is not guaranteed or endorsed by the publisher.

## References

[1] Centers for disease control and prevention. Understanding the Epidemic. (2020). Available online at: https://www.cdc.gov/drugoverdose/epidemic/index.html

[2] Carroll KM. Lost in translation? moving contingency management and cognitive behavioral therapy into clinical practice. Ann N Y Acad Sci. (2014) 1327:94–111. 10.1111/nyas.12501 25204847 PMC4206586

[3] Madson M Schumacher J Baer J Martino S. Motivational interviewing for substance use: mapping out the next generation of research. J Subst Abuse Treat. (2016) 65:1–5. 10.1016/j.jsat.2016.02.003 26971078

[4] McHugh RK Hearon BA Otto MW. Cognitive behavioral therapy for substance use disorders. Psychiatr Clin North Am. (2010) 33:511–25. 10.1016/j.psc.2010.04.012 20599130 PMC2897895

[5] Ramsay J. CBT for Adult ADHD: adaptations and hypothesized mechanisms of change. J Cogn Psychother. (2010) 24:37–45. 10.1891/0889-8391.24.1.37

[6] Jhanjee S. Evidence based psychosocial interventions in substance use. Indian J Psychol Med. (2014) 36:112–8. 10.4103/0253-7176.130960 24860208 PMC4031575

[7] Rapeli P Kivisaari R Autti T Kahkonen S Puuskari V Jokela O. Cognitive function during early abstinence from opioid dependence: a comparison to age, gender, and verbal intelligence matched controls. BMC Psychiatry. (2006) 6:9. 10.1186/1471-244X-6-9 16504127 PMC1489929

[8] Verdejo-García A López-Torrecillas F Giménez C Pérez-García M. Clinical implications and methodological challenges in the study of the neuropsychological correlates of cannabis, stimulant, and opioid abuse. Neuropsychol Rev. (2004) 14:1–41. 10.1023/B:NERV.0000026647.71528.83 15260137

[9] Schiltenwolf M Akbar M Hug A Pfuller U Gantz S Neubauer E. Evidence of specific cognitive deficits in patients with chronic low back pain under long-term substitution treatment of opioids. Pain Physician. (2014) 17:9–20. 10.36076/ppj.2014/17/9 24452649

[10] Anand P Springer SA Copenhaver MM Altice FL. Neurocognitive impairment and HIV risk factors: a reciprocal relationship. AIDS Behav. (2010) 14:1213–26. 10.1007/s10461-010-9684-1 20232242 PMC2906682

[11] Baldacchino A Balfour DJK Passetti F Humphris G Matthews K. Neuropsychological consequences of chronic opioid use: a quantitative review and meta-analysis. Neurosci Biobehav Rev. (2012) 36:2056–68. 10.1016/j.neubiorev.2012.06.006 22771335

[12] Altice FL Kamarulzaman A Soriano VV Schechter M Friedland GH. Treatment of medical, psychiatric, and substance-use comorbidities in people infected with HIV who use drugs. Lancet. (2010) 376:367–87. 10.1016/S0140-6736(10)60829-X 20650518 PMC4855280

[13] Kamarulzaman A Altice FL. Challenges in managing HIV in people who use drugs. Curr Opin Infect Dis. (2015) 28:10–6. 10.1097/QCO.0000000000000125 25490106 PMC4409950

[14] Huedo-Medina TB Shrestha R Copenhaver M. Modeling a theory-based approach to examine the influence of neurocognitive impairment on HIV risk reduction behaviors among drug users in treatment. AIDS Behav. (2016) 20:1646–57. 10.1007/s10461-016-1394-x 27052845 PMC4945437

[15] Fishbein DH Krupitsky E Flannery BA Langevin DJ Bobashev G Verbitskaya E. Neurocognitive characterizations of Russian heroin addicts without a significant history of other drug use. Drug Alcohol Depend. (2007) 90:25–38. 10.1016/j.drugalcdep.2007.02.015 17382488 PMC1991277

[16] Verdejo-García A Pérez-García M. Profile of executive deficits in cocaine and heroin polysubstance users: common and differential effects on separate executive components. Psychopharmacology. (2007) 190:517–30. 10.1007/s00213-006-0632-8 17136401

[17] McDonald S Darke S Kaye S Torok M. Deficits in social perception in opioid maintenance patients, abstinent opioid users and non-opioid users. Addiction. (2013) 108:566–74. 10.1111/add.12040 23164063

[18] Weiss NH Kiefer R Goncharenko S Raudales AM Forkus SR Schick MR. Emotion regulation and substance use: a meta-analysis. Drug Alcohol Depend. (2022) 230:109131. 10.1016/j.drugalcdep.2021.109131 34864568 PMC8714680

[19] Rezapour T Hatami J Farhoudian A Sofuoglu M Noroozi A Daneshmand R. Cognitive rehabilitation for individuals with opioid use disorder: a randomized controlled trial. Neuropsychol Rehabil. (2019) 29:1273–89. 10.1080/09602011.2017.1391103 29161998

[20] American Society of Addictive Medicine Mee-Lee D. The ASAM Criteria: Treatment Criteria for Addictive, Substance-Related, and Co-Occurring Conditions. 3rd ed. The Change Companies (2013).

[21] National Institute of Health. Cognition Measures. Available online at: https://www.healthmeasures.net/explore-measurement-systems/nih-toolbox/intro-to-nih-toolbox/cognition (accessed December 9, 2020).

[22] Copenhaver MM Sanborn V Shrestha R Mistler C Gunstad J. Association between the brief inventory of neurocognitive impairment (BINI) and objective cognitive testing among persons with opioid use disorders in drug treatment. J Addict Dis. (2020) 39:1–9. 10.1080/10550887.2020.1831129 33047651 PMC11395862

[23] Simon SS Yokomizo JE Bottino CM. Cognitive intervention in amnestic Mild Cognitive impairment: a systematic review. Neurosci Biobehav Rev. (2012) 36:1163–78. 10.1016/j.neubiorev.2012.01.007 22322184

[24] Tomaszewski Farias S Schmitter-Edgecombe M Weakley A Harvey D Denny KG Barba C. Compensation strategies in older adults: association with cognition and everyday function. Am J Alzheimers Dis Other Demen. (2018) 33:184–91. 10.1177/1533317517753361 29357670 PMC10852491

[25] Barman A Chatterjee A Bhide R. Cognitive impairment and rehabilitation strategies after traumatic brain injury. Indian J Psychol Med. (2016) 38:172–81. 10.4103/0253-7176.183086 27335510 PMC4904751

[26] Gallagher M McLeod H McMillan T. A systematic review of recommended modifications of CBT for people with cognitive impairments following brain injury. Neuropsychol Rehabil. (2017) 29:1–21. 10.1080/09602011.2016.1258367 27873549

[27] Ezeabogu I Copenhaver MM Potrepka J. The influence of neurocognitive impairment on HIV treatment outcomes among drug-involved people living with HIV/AIDS. AIDS Care. (2012) 24:386–93. 10.1080/09540121.2011.608794 22250847 PMC3294373

[28] Krueger RA. Focus Groups: A Practical Guide For Applied Research / by Richard A. Krueger, TX: Sage Publications, Inc. (1989).

[29] Ruff CC Alexander IM McKie C. The use of focus group methodology in health disparities research. Nurs Outlook. (2005) 53:134–40. 10.1016/j.outlook.2005.03.010 15988450

[30] Centers for Disease Control and Prevention. Data Collection Methods for Program Evaluation: Focus Groups (2018).

[31] Carlsen B Glenton C. What about N? a methodological study of sample-size reporting in focus group studies. BMC Med Res Methodol. (2011) 11:26. 10.1186/1471-2288-11-26 21396104 PMC3061958

[32] Fisher JD Cornman DH Osborn CY Amico KR Fisher WA Friedland GA. Clinician-initiated HIV risk reduction intervention for HIV-positive persons: formative research, acceptability, and fidelity of the options project. J Acquir Immune Defic Syndr. (2004) 37:S78–87. 10.1097/01.qai.0000140605.51640.5c 15385903

[33] Vissman AT Hergenrather KC Rojas G Langdon SE Wilkin AM Rhodes SD. Applying the theory of planned behavior to explore HAART adherence among HIV-positive immigrant Latinos: elicitation interview results. Patient Educ Couns. (2011) 85:454–60. 10.1016/j.pec.2010.12.004 21208772

[34] Glaser B Strauss A. The discovery of grounded theory: strategies for qualitative research. Sociology Press. (1967) 17:364. 10.1097/00006199-196807000-00014

[35] Timmermans S Tavory I. Theory construction in qualitative research: from grounded theory to abductive analysis. Sociological Theory. (2012) 30:167–86. 10.1177/0735275112457914

[36] Deterding N Waters M. Flexible coding of in-depth interviews: a twenty-first-century approach. Sociol Methods Res. (2018) 50:004912411879937. 10.1177/0049124118799377

[37] QSR International Pty Ltd. NVivo (2021).

[38] Kidd PS Parshall MB. Getting the focus and the group: enhancing analytical rigor in focus group research. Qual Health Res. (2000) 10:293–308. 10.1177/104973200129118453 10947477

[39] Saldaña J. The Coding Manual for Qualitative Research. Thousand Oaks, CA: Sage Publications (2009).

[40] Jootun D McGhee G Marland GR. Reflexivity: promoting rigour in qualitative research. Nurs Stand. (2009) 23:42–6. 10.7748/ns2009.02.23.23.42.6800 19263905

[41] Gwadz MV Collins LM Cleland CM Leonard NR Wilton L Gandhi M. Using the multiphase optimization strategy (MOST) to optimize an HIV care continuum intervention for vulnerable populations: a study protocol. BMC Public Health. (2017) 17:383. 10.1186/s12889-017-4279-7 28472928 PMC5418718

[42] Centers for disease control and prevention. Opioid Overdose. Available online at: https://www.cdc.gov/drugoverdose/data/otherdrugs.html#:~:text=Polysubstance%20drug%20use%20occurs%20withor%20other%20non%2Dopioid%20substances (accessed July 1, 2020).

[43] Centers for disease control and prevention. Drug Overdose Deaths. Available online at: https://www.cdc.gov/drugoverdose/data/statedeaths.html (accessed May 25, 2020).

[44] Centers for disease control and prevention. Drug Overdose Deaths in the U.S. Top 100,000 Annually (2021).

[45] Centers for disease control and prevention. HIV and People Who Inject Drugs. Available online at: https://www.cdc.gov/hiv/group/hiv-idu.html (accessed September 29, 2020).

